# Circulating Microbiota-Based Metagenomic Signature for Detection of Hepatocellular Carcinoma

**DOI:** 10.1038/s41598-019-44012-w

**Published:** 2019-05-17

**Authors:** Eun Ju Cho, Sangseob Leem, Sun Ah Kim, Jinho Yang, Yun Bin Lee, Soon Sun Kim, Jae Youn Cheong, Sung Won Cho, Ji Won Kim, Sung-Min Kim, Jung-Hwan Yoon, Taesung Park

**Affiliations:** 1 Department of Internal Medicine and Liver Research Institute, Seoul National University College of Medicine, Seoul, Korea; 2 Department of Statistics, Seoul National University, Seoul, Korea; 3 Department of Health and Safety Convergence Science, Korea University, Seoul, Korea; 4 Department of Gastroenterology, Ajou University School of Medicine, Suwon, Korea; 5 Department of Internal Medicine, Seoul National University Boramae Hospital, Seoul National University College of Medicine, Seoul, Korea; 6 Department of Internal Medicine, Inje University Haeundae Paik Hospital, Busan, Korea

**Keywords:** Hepatocellular carcinoma, Tumour biomarkers

## Abstract

Circulating microbial dysbiosis is associated with chronic liver disease including nonalcoholic steatohepatitis and alcoholic liver disease. In this study, we evaluated whether disease-specific alterations of circulating microbiome are present in patients with cirrhosis and hepatocellular carcinoma (HCC), and their potential as diagnostic biomarkers for HCC. We performed cross-sectional metagenomic analyses of serum samples from 79 patients with HCC, 83 with cirrhosis, and 201 matching healthy controls, and validated the results in the same number of subjects. Serum bacterial DNA was analyzed using high-throughput pyrosequencing after amplification of the V3–V4 hypervariable regions of 16S rDNA. Blood microbial diversity was significantly reduced in HCC, compared with cirrhosis and control. There were significant differences in the relative abundances of several bacterial taxa that correlate with the presence of HCC, thus defining a specific blood microbiome-derived metagenomic signature of HCC. We identified 5 microbial gene markers-based model which distinguished HCC from controls with an area under the receiver-operating curve (AUC) of 0.879 and a balanced accuracy of 81.6%. In the validation, this model accurately distinguished HCC with an AUC of 0.875 and an accuracy of 79.8%. In conclusion, circulating microbiome-based signatures may be potential biomarkers for the detection HCC.

## Introduction

Gut microbiota dysbiosis and increased bacterial translocation play an important role in the progression of chronic liver disease. Because liver receive most of the blood supply from intestine, it is exposed to gut microbiota, bacterial pathogen-associated molecular patterns and microbial metabolites which lead to chronic inflammation and progression of liver disease such as fibrosis and hepatocellular carcinoma (HCC)^1,2^.

Recent studies have reported the presence of circulating bacterial contents in healthy human blood by sequencing 16S ribosomal deoxyribonucleic acid (rDNA) genes^3^. Furthermore, the predictive roles of circulating microbiome on the onset of diabetes and cardiovascular events have been suggested in longitudinal studies^4,5^. In addition, changes in blood- and gut-microbiome signatures have been linked to liver fibrosis in obese patients^6^. Moreover, changes in blood-microbiome signatures associated with a shift in the metabolic functions has been reported in heavy alcohol drinkers without significant liver disease and those with alcoholic hepatitis^7^. Although it has not been known yet whether alteration in the blood microbiome is just a bystander of dysbiosis or a true player in the pathophysiology of disease, these findings collectively suggest that blood microbiota profiles might be used as a potential noninvasive biomarker. However, to our knowledge, the association of blood microbiota and HCC has not been studied, yet.

In this study, we evaluated whether disease-specific microbiome alterations are present in the blood microbiome from patients with cirrhosis and HCC, and their potential as diagnostic biomarkers for HCC.

## Materials and Methods

### Study population

The study population consisted of 158 patients with HCC and 166 with cirrhosis diagnosed between July 2013 and August 2017, at the Seoul National University Hospital (Seoul, Korea) and Ajou University Hospital (Suwon, Korea), and 402 healthy controls who underwent a medical checkup at the Seoul National University Boramae Medical Center (Seoul, Korea) and the Inje University Haeundae Paik Hospital (Busan, Korea) during the same period. Subjects within each hospital were randomly divided in a 1:1 ratio to a model development set and a test set (79 HCC, 83 cirrhosis and 201 controls for each set) to develop a model capable of distinguishing HCC from cirrhosis and controls.

The diagnosis of HCC was mainly based on the noninvasive criteria of the international guidelines^8,9^. Cirrhosis was diagnosed histologically, clinically, or by typical radiological findings^10^. The control group was defined by subjects with no clinical or imaging evidence of liver disease. Subjects with clinical symptoms and signs of infection were excluded from the study. Clinical data were collected using standard case report forms. Overnight fasting serum samples were obtained from all subjects before any treatment using standard protocols and stored at −80 °C until processing.

### Ethics approval and consent to participate

This study was conducted in accordance with the Declaration of Helsinki principles. It was approved by the institutional review board (IRB) of Seoul National University Hospital (IRB No. 1704-021-843), and samples derived from the Korea Biobank Network were obtained with informed consent under IRB-approved protocols. The Reporting Recommendations for Tumor Marker Prognostic Studies criteria were followed throughout the study^11^.

### Circulating cell-free DNA extraction from serum samples

Serum samples were centrifuged at 2,000 g for 15 min at 4 °C to remove cell debris, and the supernatant was collected and incubated with proteinase K at 56 °C for 30 minutes. Then, the samples were boiled for 40 min under 100 °C to extract DNA out of extracellular vesicles, and the supernatant was collated by centrifugation for 30 min at 10,000 g under 4 °C. Total DNA was extracted from the supernatant (1 mL) using a DNA isolation kit (DNeasy Blood & Tissue Kit, QIAGEN, Germany) and quantified by using the QIAxpert system (QIAGEN, Germany).

### Metagenomic sequencing and annotation

Following DNA extraction, bacterial genomic DNA was amplified with 16S_V3_F (5′-TCGTCGGCAGCGTCAGATGTGTATAAGAGACAGCCTACGGGNGGCWGCAG-3′) and 16S_V4_R (5′-GTCTCGTGGGCTCGGAGATGTGTATAAGAGACAGGACTACHVGGGTATCTAATCC-3′) primers, which are specific for V3-V4 hypervariable regions of 16S rDNA. The libraries were prepared using PCR products according to MiSeq System guide (Illumina, USA) and quantified using a QIAxpert (QIAGEN, Germany). Each amplicon is then quantified and sequenced on a MiSeq (Illumina, USA) according to the manufacturer’s recommendations.

### Profiling

Raw sequencing reads obtained from the sequencer were filtered according to the barcode and primer sequences using MiSeq (Illumina, USA). Taxonomic assignment was performed by profiling program MDx-Pro ver.1. The high-quality sequencing reads were selected after checking the read length (≥300 bp) and the quality score (average Phred score ≥ 20). Operational Taxonomy Units (OTUs) were clustered by using sequence clustering algorithms CD-HIT. Subsequently, taxonomy assignment was carried out by using UCLUST and QIIME against the Greengenes reference database (gg_13_5_99)^12,13^. OTUs with a number of sequences <0.005% of the total were removed from the OTU table. After filtering, an average of 14,555 reads per sample was obtained (min: 19; max: 31139) and sample with low number of read counts (<2500) were filtered out for quality controls. The resulting OTU table was used for predictive functional analysis using the software Tax4Fun in the package metagenomics version 0.1.0^14^.

### Statistical analysis

All statistical analyses were performed with R version 3.4.4 on Windows 10 (Version 3.4.4, http://www.R-project.org). For significance tests for the difference among the three groups, one-way analysis of variance was used for continuous variables, and the chi-square test was used for categorical variables. α-diversity of microbiota for each sample was measured by Shannon index. To compare α-diversities between groups, the Wilcoxon rank-sum test was used for comparing two groups and the Kruskal-Wallis test was used for more than two groups. β-diversity of a pair of samples was measured by weighted and unweighted UniFrac distances^15,16^. Based on the UniFrac distances, principal coordinate analysis (PCoA) was performed and analysis of similarity (ANOSIM) permutation test was used to assess the statistical significance of the separation among groups^17^. The OTUs after removing the unassigned ones at phylum level were used to calculate the relative abundance for comparisons of groups and model construction.

To develop a model for discriminating HCC, we used the logistic regression model in which the response variable is a binary variable distinguishing HCC and control groups. We selected OTUs with significant effects in logistic regression model that consists of binary response variable and age/sex as adjustment variables.

We first randomly divided data into the model development and test sets. Data of the model development set were further randomly allocated into training and validation sets. As the rare OTUs differed significantly according to test methods and the relatively abundant OTUs were similar across the methods^18^, we selected candidate OTUs at genus level if their means of relative abundance were greater than 1% and if the *p* values were smaller than 0.05 in logistic regression models with age and sex as covariates. For abundance comparisons of OTUs between groups, *p* values are adjusted by Bonferroni correction based on the number of OTUs after filtering. Then all possible combinations of candidate OTUs were tested by repeating 10 times for two-fold cross validations to find the optimal model discriminating HCC. The final model was selected by the one with the lowest Akaike’s information criteria (AIC) from the model development set, and then its performance was assessed using the test set.

## Results

### Baseline characteristics

The baseline characteristics of subjects in the model development and test sets are described in Table 1. Age and gender were comparable between three groups in both sets. Most of the patients in HCC group had hepatitis virus-related compensated liver disease, whereas cirrhosis group showed relatively higher proportion of decompensated, non-viral etiology (mainly alcohol)-induced cirrhosis. In both sets, about 80% of the HCC cases belonged to stage I and II according to the 7th American Joint Committee on Cancer staging system.

**Table 1:** Baseline characteristics of the model development and test sets.

	Development set			*p*	Test set			*p*
	HCC (n = 79)	Cirrhosis (n = 83)	Healthy controls (n = 201)		HCC (n = 79)	Cirrhosis (n = 83)	Healthy controls (n = 201)	
Age, years	58.6 ± 9.6	57.1 ± 10.7	57.6 ± 10.4	0.61	58.8 ± 10.3	56.5 ± 9.9	56.6 ± 10.0	0.22
Male	58 (73.4%)	62 (74.7%)	119 (71.2%)	0.83	64 (81.0%)	58 (70.0%)	127 (75.1%)	0.26
**Etiology of liver disease**								
Viral	73 (92.4%)	54 (65.1%)	—	<0.001	70 (88.6%)	71 (85.5%)	—	0.73
Non-viral	6 (8.6%)	29 (34.9%)	—		9 (11.3%)	12 (14.5%)	—	
**Liver function**								
Compensated	71 (89.9%)	56 (67.5%)	—	0.001	72 (91.1%)	62 (73.5%)	—	0.006
Decompensated	8 (10.1%)	27 (32.5%)	—		7 (8.9%)	22 (26.5%)	—	
α-fetoprotein, ng/mL, median (IQR)	4.8 (2.6–7.4)	11.1 (4.6–71.2)	—	<0.001	3.7 (2.2–11.8)	13.5 (4.5–103.4)	—	<0.001
**AJCC TNM stage, 7** ^**th**^								
I	43 (54.4%)		—		36 (45.6%)		—	
II	23 (29.1%)		—		25 (31.6%)		—	
III	9 (11.4%)		—		10 (12.7%)		—	
IV	4 (5.1%)		—		8 (10.1%)		—	

Data are presented as means with standard deviation (SD) or numbers (%), unless otherwise indicated.

### Changes in the taxonomic signature of blood metagenomes according to liver disease

**Figure 1:**
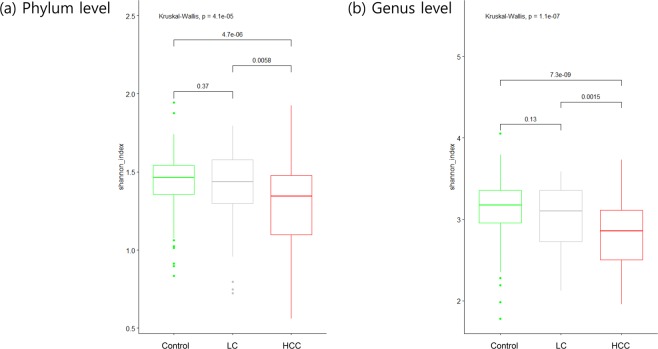
Reduced blood microbial diversities in the hepatocellular carcinoma (n = 79) and cirrhosis groups (n = 83) compared with control group (n = 201). α-diversity (Shannon index) of the three groups (**a**) at the phylum and (**b**) at the genus levels. *p* values from Kruskal–Wallis tests are shown.

**Figure 2:**
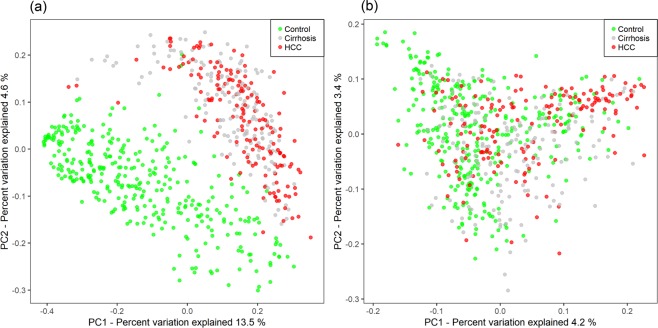
Differences of microbiome between the three groups. The PCoA plots based on (**a**) unweighted UniFrac distance and (**b**) weighted UniFrac distance. The control samples are colored as green, cirrhosis as gray, and HCC samples as red.

To investigate whether there are specific changes of blood metagenomes according to the liver disease, we assessed the relative abundance of taxa in the three group. We used the Shannon index at each level to compare α-diversity (within sample diversity). At the phylum level, α-diversity was significantly reduced in the HCC, compared with the cirrhosis and control groups (*p* = 0.006 and 4.7e-06, respectively), whereas there was no significant difference between the cirrhosis and control groups (*p* = 0.37; Fig. 1a). Similar trends were also observed at the genus level (Fig. 1b). To compare β-diversity (between sample diversity), we used the unweighted and weighted UniFrac distances. The PCoA plot based on the unweighted UniFrac distance showed the strong separation between control group and cirrhosis and HCC groups (Fig. 2a). The ANOSIM test confirmed the significance (control *vs*. cirrhosis: R = 0.45, *p* < 0.001; control *vs*. HCC: R = 0.49, *p* < 0.001; cirrhosis *vs*. HCC: R = 0.04, *p* < 0.001). While no clear separation was observed in the PCoA plot based on the weighted UniFrac distance (Fig. 2b), the ANOSIM test provided significant differences between groups (control *vs*. cirrhosis: R = 0.20, *p* < 0.001; control *vs*. HCC: R = 0.19, *p* < 0.001; cirrhosis *vs*. HCC: R = 0.04, *p* < 0.001). Compared to the unweighted UniFrac distance, the weighted UniFrac distance yielded much smaller R values. These results suggest that there are some microbiome factors that could distinguish the HCC group from the others.

### Blood taxonomic signature characterizing patients with HCC in the development cohort

**Figure 3:**
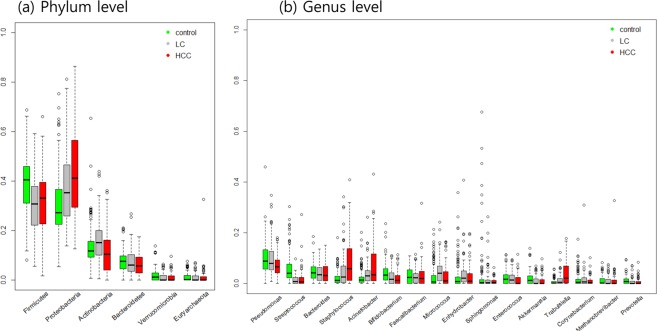
Relative abundances of abundant OTUs among the three groups. Boxplots of the relative abundances of abundant OTUs (**a**) at phylum and (**b**) at the genus levels in the three groups. Abundant OTUs are selected by relative abundance means of OTUs > 1% in total samples and listed by decreasing order of the mean values.

Next, we investigated abundant OTUs defined as mean relative abundances of OTUs >1% in total samples at phylum and genus levels. Relative abundances of OTUs are grouped for disease groups and plotted by the decreasing order of mean relative abundances of total samples in Fig. 3. The blood microbiomes in three groups were dominated by members of Firmicutes and Proteobacteria, followed by Actinobacteria and Bacteroidetes in much lower abundances. In addition, both Firmicutes and Proteobacteria were differentially abundant across the three groups (*p* < 0.05), with Firmicutes being highest in controls while Proteobacteria was highest in HCC group.

At the genus level, 7 bacterial taxa showed significantly different abundance between HCC and control groups (*p* < 0.05, multiple testing correction using Bonferroni, univariate test with adjustment of age and sex). *Pseudomonas* was the most abundant microbiome in the three groups, and significantly decreased in HCC, compared with control group. Of the remaining 6 taxa, *Streptococcus* and *Bifidobacterium* were significantly increased in controls, whereas 4 taxa including *Staphylococcus*, *Acinetobacter*, *Klebsiella* and *Trabulsiella* were significantly enriched in HCC-associated microbiomes. Remarkably, *Staphylococcus* showed the strongest association with HCC (*p* = 4.0e-08) and demonstrated a 4.3-fold increase in HCC, compared with controls.

We evaluated the effects of liver function and etiology of liver disease on the relative abundance of blood microbiome. When patients with cirrhosis and/or HCC were stratified into compensated and decompensated stages, there was no significant difference in α-diversity (supplementary Fig. S1A). However, the patterns of β-diversity provided some evidence of differences according to liver function (compensated *vs*. decompensated: weighted UniFrac ANOSIM, R = 0.06, *p* = 0.046; Unweighted UniFrac ANOSIM, R = 0.06, *p* = 0.06; supplementary Fig. S1B). On the contrary, there was no significant difference in α-diversity (supplementary Fig. S2A) and β-diversity according to the etiology of liver disease (viral *vs*. non-viral: weighted UniFrac ANOSIM, R = 0.02, *p* = 0.30; Unweighted UniFrac ANOSIM, R = 0, *p* = 0.49; supplementary Fig. S2B).

### HCC discrimination with the 5-genera microbiome signature

**Figure 4:**
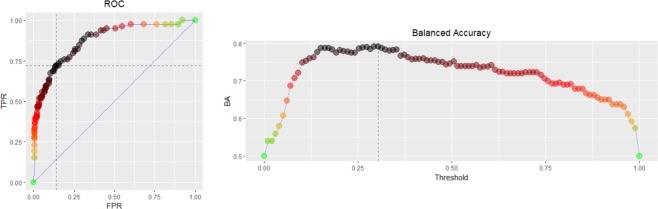
Receiver operating characteristic curve of 5-genera signature for the test set.

To evaluate the potential of blood taxonomic signature to discriminate HCC from controls, all combination models of candidate OTUs, which are selected at the genus level (supplementary Table 1), were tested with adjustment for age and sex. Table 2 showed performance of top models for each number of microbiome markers, and the model composed of 5 HCC-associated genera (i.e., *Pseudomonas*, *Streptococcus*, *Staphylococcus*, *Bifidobacterium*, and *Trabulsiella*) was finally selected. The model based on these five OTUs showed an area under the receiver-operating curve (AUC) value of 0.879 (sensitivity, 0.729; specificity, 0.850; accuracy, 0.816) in the model development set. Subsequently, we evaluated its performance in the test set. The AUC was 0.875 (sensitivity, 0.756; specificity, 0.797; accuracy, 0.798; Fig. 4), suggesting a potential for the blood microbiome-based signature to accurately discriminate HCC from controls. When the model was applied to three groups, the probability of disease was significantly increased in the HCC group versus the control group, and the cirrhosis group was intermediate level between the HCC and control group both in the model development set and test set (supplementary Fig. S3). In addition, these trends significantly maintained regardless of underlying liver function status, suggesting that the changes from healthy control to cirrhosis and further to HCC might be correlated with the disease progression status, rather than liver function status. In addition, we performed additional 5-fold and 10-fold cross validations and compared their performances. We found that high number of folds increases model performances (i.e. sensitivity, specificity, accuracy and AUC) significantly which might be due to a small sample size (data not shown). To provide a more conservative (i.e. smaller AUC values) results, we decided to present the 2-fold cross validation results.

**Table 2:** Performance of top models for each number of microbiome markers.

Number of microbiome markers	Model string	AIC	Training				Validation				AUC in testing
			Sensitivity	Specificity	Accuracy	AUC	Sensitivity	Specificity	Accuracy	AUC	
10	class~age + sex + g778 + g362 + g319 + g769 + g147 + g725 + g348 + g837 + g732 + g179	208.8	0.845	0.865	0.859	0.923	0.727	0.834	0.804	0.860	0.893
9	class~age + sex + g778 + g362 + g319 + g147 + g725 + g348 + g837 + g732 + g179	207.1	0.844	0.856	0.853	0.920	0.750	0.838	0.813	0.873	0.892
8	class~age + sex + g778 + g362 + g319 + g147 + g725 + g837 + g732 + g179	206.4	0.844	0.859	0.855	0.921	0.748	0.836	0.811	0.865	0.887
7	class~age + sex + g778 + g362 + g319 + g147 + g725 + g837 + g732	205.9	0.814	0.856	0.844	0.914	0.759	0.861	0.832	0.886	0.885
6	class~age + sex + g778 + g362 + g319 + g147 + g837 + g732	205.3	0.808	0.863	0.848	0.916	0.746	0.833	0.808	0.870	0.887
5*	**class~age + sex** + **g778 + g362 + g319** + **g147 + g732**	**205.1**	**0.797**	**0.871**	**0.850**	**0.908**	**0.729**	**0.850**	**0.816**	**0.879**	**0.875**
4	class~age + sex + g362 + g319 + g147 + g732	206.3	0.783	0.855	0.835	0.904	0.746	0.836	0.811	0.872	0.875
3	class~age + sex + g362 + g147 + g732	211.9	0.804	0.830	0.823	0.901	0.757	0.829	0.809	0.877	0.850
2	class~age + sex + g362 + g732	221.6	0.790	0.823	0.814	0.888	0.761	0.790	0.781	0.864	0.820
1	class~age + sex + g319	257.5	0.624	0.835	0.775	0.795	0.597	0.822	0.759	0.776	0.767
0	class~age + sex	321.6	0.633	0.572	0.589	0.660	0.637	0.571	0.590	0.650	0.562

^*^The finally selected model showing the lowest AIC.

^**^The OTU symbols are as following: g778, *Pseudomonas*; g362, *Streptococcus*; g319, *Staphylococcus*; g769, *Acinetobacter*; g147, *Bifidobacterium*; g725, *Klebsiella*; g348, *Enterococcus*; g837, *Akkermansia*; g732, *Trabulsiella*; g179, *Prevotella*.

## Discussion

In this study, we investigated the relationship between circulating microbiota and HCC for the first time. The main result of this study was that HCC was associated with altered composition of circulating microbiota, as well as significant lower level of diversity. In addition, we validated the diagnostic accuracy of the blood microbiome-derived metagenomic signature to detect HCC, and suggested their potential as diagnostic markers for HCC.

A recent study has reported that the blood 16S rDNA concentration was shown to be increased in obese patients with hepatic fibrosis, whereas the bacterial diversity was decreased^6^. Another study has shown that there were multiple alterations in the circulating microbiome in heavy drinkers and patients with alcoholic hepatitis, and these alterations were associated with changes in the metabolic functions such as activation of the type III secretion system associated with gram-negative bacteria, and increased isoprenoid synthesis and anthranilate degradation which are well-known modulators of biofilm formation and gram-positive bacterial growth^7^. In our study, we showed changes in the composition of the circulating microbiota that correlate with the presence of HCC. These results indicate that specific blood dysbiosis is associated with chronic liver disease including nonalcoholic steatohepatitis, alcoholic liver disease and HCC.

Emerging studies have shown that gut microbiome can affect the pathophysiology of HCC. Gut microbiome has been known to activate lipopolysaccharide (LPS)-toll-like receptor 4 (TLR4) pathway, leading to promotion of HCC growth in mice^2,19^. Furthermore, gut microbiome–mediated bile acid metabolism modulates antitumor surveillance against HCC via hepatic natural killer T cells^20^. In addition, a recent study has reported characteristic alterations of gut microbiome in patients with early HCC, and suggested OTUs markers as a diagnostic tool for HCC^21^. These results indicate a global shift in gut microbiome in HCC, and the altered microbial community might play an important role in the development and progression of HCC. Our study showed that circulating microbiota as well as gut microbiota also presented a moderate dysbiosis in patients with HCC, and this signature might be a potential noninvasive biomarker for detecting HCC.

In this study, the relative abundance of *Bifidobacterium* genus was notably decreased in HCC. *Bifidobacterium* has been known to reinforce gut barrier function and have protective effects on liver injury^22^. In addition, it can promote anticancer immunity and enhance the efficacy of immunotherapy^23^. Moreover, probiotics has been shown to inhibit the development and growth of HCC in animal model by changing the composition of gut microbiota and recovering intestinal permeability^24,25^. *Akkermansia*, a well-known intestinal commensal promoting intestinal integrity and ameliorating liver injury^26^, also showed decreasing tendency in HCC compared with controls, even though the difference was not statistically significant. On the contrary, the relative abundance of potentially pathogenic gram-negative bacteria producing LPS, such as *Klebsiella* and *Acinetobacter*, were increased in HCC. High levels of LPS activate NF-κB pathway and enhance inflammatory damage in liver, thereby promoting the development of HCC^27^. Furthermore, LPS-TLR4 pathway promotes epithelial-mesenchymal transition, invasion and metastasis of HCC^2,28^. Collectively, the decrease of potentially beneficial bacteria protecting intestinal barrier, and the increase of potentially pathogenic bacteria might affect intestinal and hepatic inflammation, promoting the development and progression of HCC.

There are several limitations to the present study. First, although there was no subject presenting symptoms and signs of infection at the time of blood sampling, medication history was not available in the healthy control group. Therefore, those taking antibiotics or pro/prebiotics could be included and affect the results. However, the key features of the changes across groups in the development set were maintained in the test set. In addition, a previous study showed that even though antibiotic exposure altered blood microbiome, it did not affect the principal differences between alcoholic hepatitis and control^7^. Second, this study could not reveal whether blood dysbiosis is only a bystander or a true player in the pathogenesis of HCC. Further studies are warranted to assess the functional and metabolic potential of the circulating microbial communities. Third, we could not analyse gut microbiome. Blood microbiome derives probably mostly from gut microbiome; however, both differ substantially from each other which suggest that gut barrier, host immune, and liver may act as modifiers^6^. Future mechanistic studies may elucidate the cross-talk between gut and blood microbiome and their functional roles in HCC.

In conclusion, this study revealed compositional dysbiosis in circulating microbiome of patients with HCC, and their potential as diagnostic biomarkers for HCC. Further independent and larger cohort studies and functional mechanistic studies are warranted to validate the identified HCC microbial signature.

## Supplementary information

Supplementary table and figures
