# Supplementary material for: Circulating Microbiota-Based Metagenomic Signature for Detection of Hepatocellular Carcinoma
Source: Sci Rep. 2019 May 17;9:7536. doi: 10.1038/s41598-019-44012-w (PMC6525191; doi:10.1038/s41598-019-44012-w)
Supplement: Supplementary file 1 — Supplementary table and figures [file 41598_2019_44012_MOESM1_ESM.pdf]

Supporting information

Circulating Microbiota-Based Metagenomic Signature for Detection of Hepatocellular Carcinoma

Eun Ju Cho[1*], Sangseob Leem[2*], Sunah Kim[2], Jinho Yang[3], Yun bin Lee[1], Soon Sun Kim[4], Jae Youn Cheong[4], Sung Won Cho[4], Ji Won Kim[5], Sung-Min Kim[6], Jung-Hwan Yoon[1**], and Taesung Park[2**]

[1]*Department of Internal Medicine and Liver Research Institute, Seoul National University College of Medicine, Seoul, Korea*

[2]*Department of Statistics, Seoul National University, Seoul, Korea*

[3]*Department of Health and Safety Convergence Science, Korea University, Seoul, Korea*

[4]*Department of Gastroenterology, Ajou University School of Medicine, Suwon, Korea*

[5]*Department of Internal Medicine, Seoul National University Boramae Hospital, Seoul National University College of Medicine, Seoul, Korea*

[6]*Department of Internal Medicine, Inje University Haeundae Paik Hospital*

*These authors equally contributed to this work.

**These co-corresponding authors equally contributed to this work.

Supplementary Table 1. Taxonomic composition used in the diagnostic models of table 2

OTU symbol	Genus	Control	Cirrhosis	HCC	*p* value
g778	Pseudomonas	10.12% (7.22%)	9.59% (6.17%)	7.03% (4.14%)	0.0097
g362	Streptococcus	5.80% (5.40%)	1.37% (1.73%)	1.93% (3.76%)	1.7e-06
g319	Staphylococcus	1.99% (2.46%)	5.04% (7.18%)	8.64% (8.98%)	4.0e-08
g769	Acinetobacter	2.17% (2.83%)	3.93% (4.38%)	6.90% (8.10%)	5.7e-06
g147	Bifidobacterium	4.03% (3.68%)	2.52% (2.78%)	2.07% (2.68%)	0.0016
g725	Klebsiella	2.02% (2.97%)	2.11% (4.23%)	4.83% (5.91%)	0.0020
g348	Enterococcus	2.21% (2.49%)	2.10% (2.40%)	1.51% (2.31%)	0.5927
g837	Akkermansia	1.85% (2.33%)	1.11% (1.65%)	1.03% (1.89%)	0.2725
g732	Trabulsiella	0.56% (0.95%)	1.43% (2.18%)	3.85% (4.46%)	2.0e-07
g179	Prevotella	1.13% (1.44%)	0.65% (1.34%)	0.69% (1.57%)	0.6813

Median and SDs of relative abundances of 10 genus used in the diagnostic models. *Significant *p* value (HCC vs healthy control with adjustment of age and sex covariates) after multiple test correction. All *p*-values of OTUs before multiple test corrections are lower than 0.05.

Supplementary Figure 1. (a) α-diversity (Shannon index) of taxa according to liver function at the phylum and genus levels. *p* values from the Kruskal–Wallis tests are shown. (b) The PCoA plot based on the unweighted and weighted UniFrac distances. The control samples are colored as green, compensated group as gray, and decompensated group as red.

(a)

Phylum level

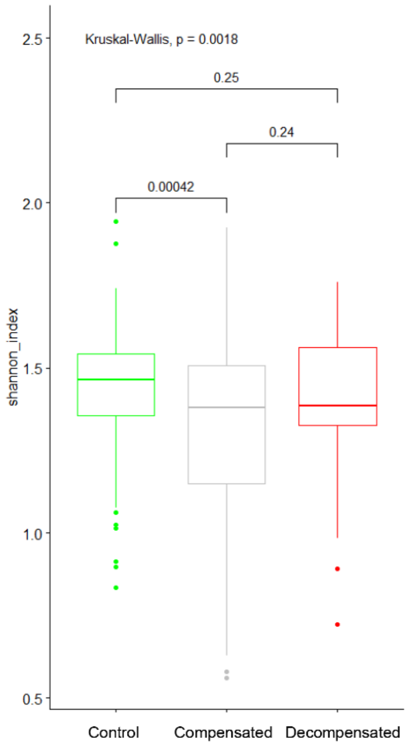

Genus level

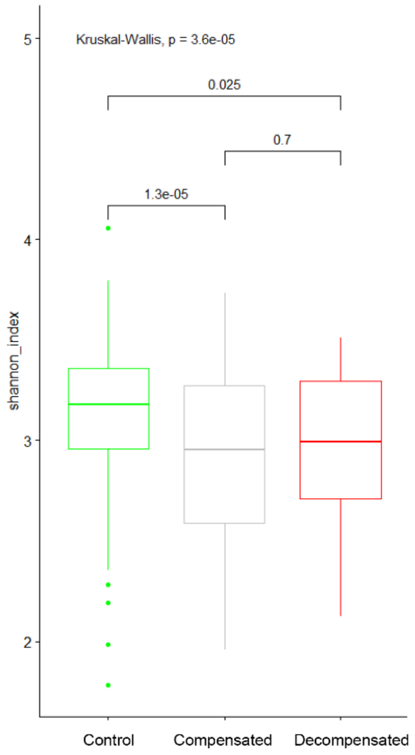

(b)

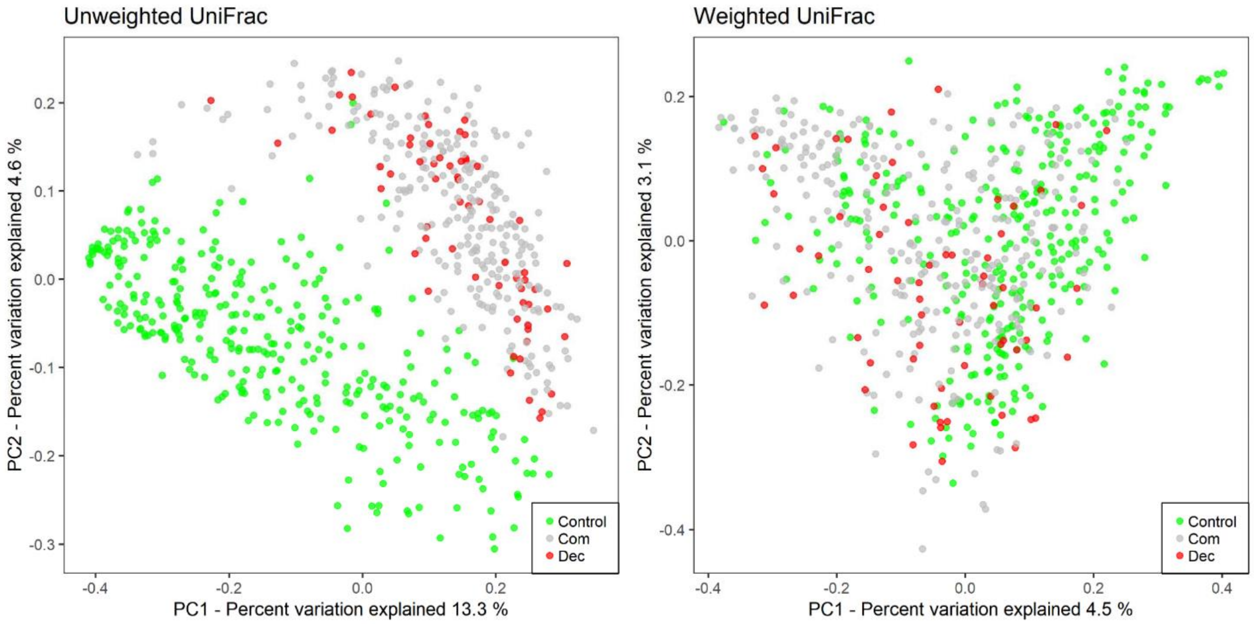
Unweighted UniFrac
PC2 - Percent variation explained 4.6 %
PC1 - Percent variation explained 13.3 %
0.2
0.1
0.0
-0.1
-0.2
-0.3
-0.4
-0.2
0.0
0.2
Control
Com
Dec
Weighted UniFrac
PC2 - Percent variation explained 3.1 %
PC1 - Percent variation explained 4.5 %
0.2
0.0
-0.2
-0.4
-0.4
-0.2
0.0
0.2
0.4
Control
Com
Dec

Supplementary Figure 2. (a) α-diversity (Shannon index) of taxa according to etiology of liver disease at the phylum and genus levels. *p* values from the Kruskal–Wallis tests are shown. (b) The PCoA plot based on the unweighted and weighted UniFrac distances. The control samples are colored as green, non-viral group as gray, and hepatitis virus-related group as red.

(a)

Phylum level

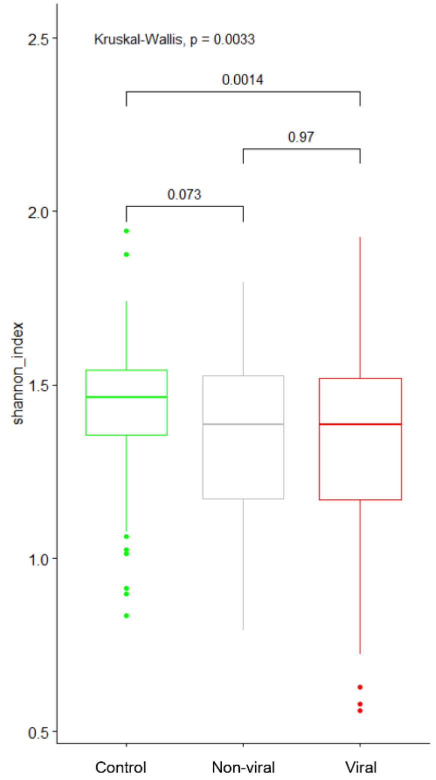

Genus level

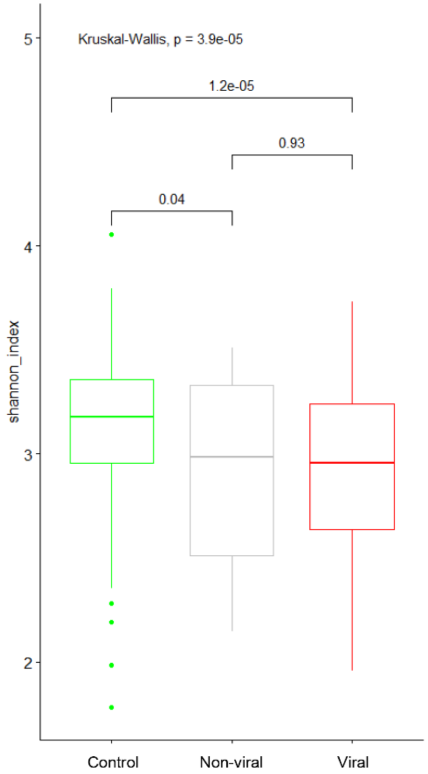

(b)

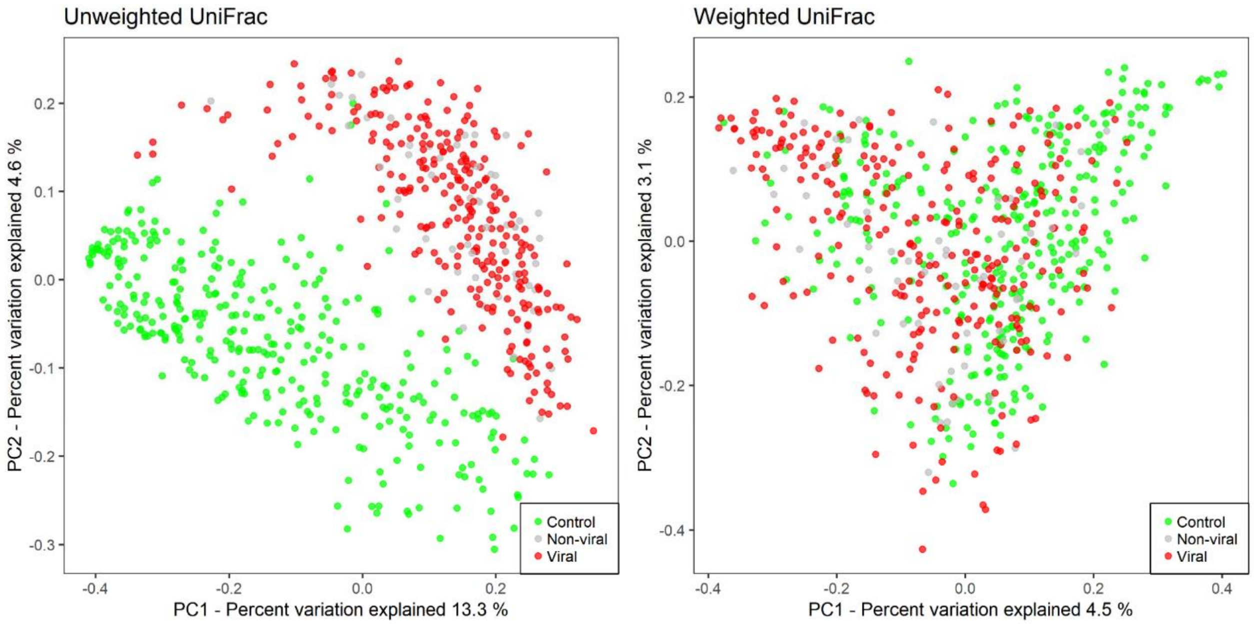
Unweighted UniFrac
PC2 - Percent variation explained 4.6 %
PC1 - Percent variation explained 13.3 %
0.2
0.1
0.0
-0.1
-0.2
-0.3
-0.4
-0.2
0.0
0.2
Control
Non-viral
Viral
Weighted UniFrac
PC2 - Percent variation explained 3.1 %
PC1 - Percent variation explained 4.5 %
0.2
0.0
-0.2
-0.4
-0.4
-0.2
0.0
0.2
0.4
Control
Non-viral
Viral

Supplementary Figure 3. The predicted probability of HCC in the three groups in the (a) model development set and (b) test set. *p* values from the Kruskal–Wallis tests or the Wilcoxon rank-sum test are shown. Com, compensated liver function, Dec, decompensated liver function, HCC, hepatocellular carcinoma

(a)

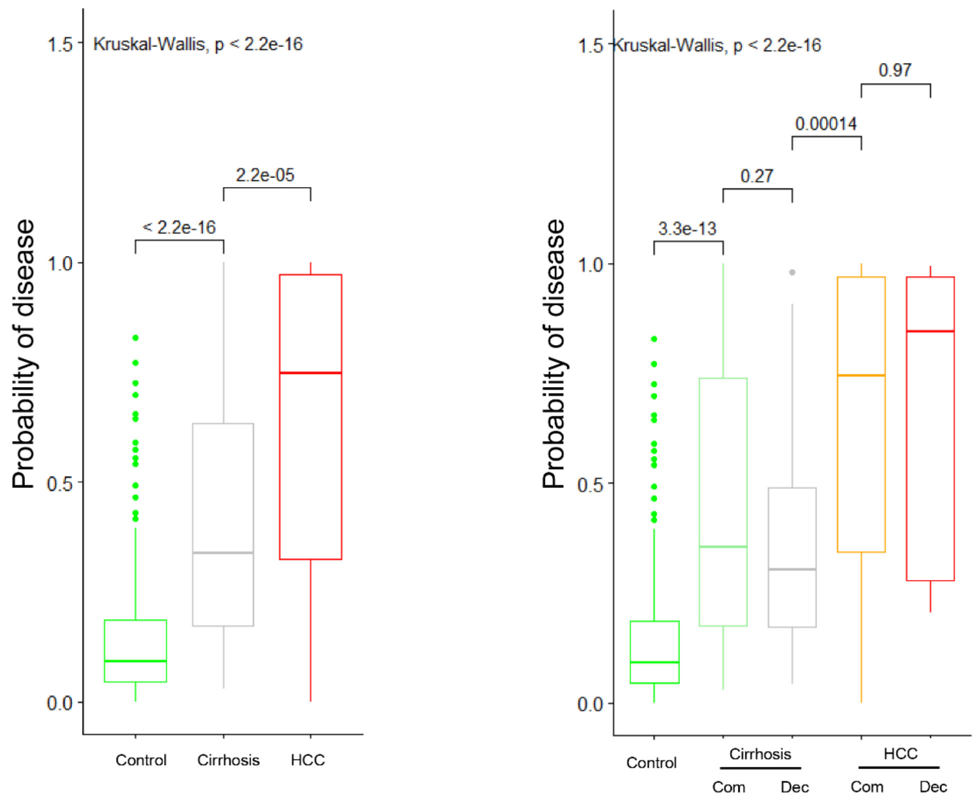

(b)

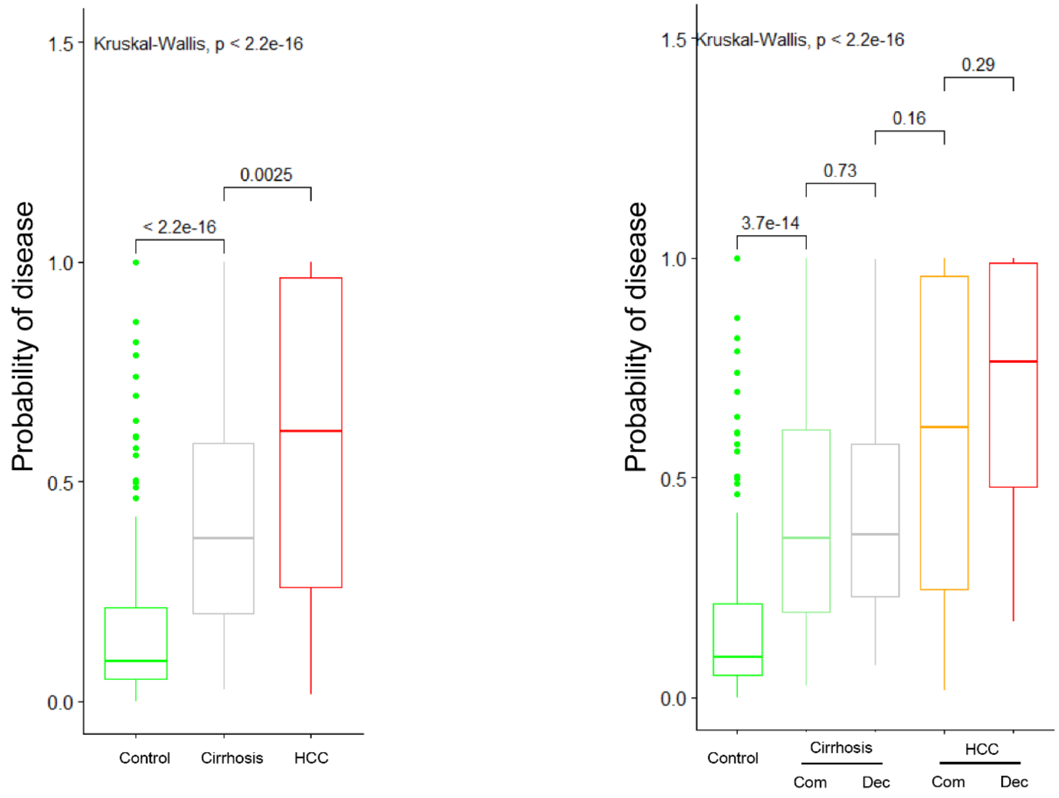

Kruskal-Wallis, p < 2.2e-16
< 2.2e-16
0.0025
Probability of disease
1.5
1.0
0.5
0.0
Control
Cirrhosis
HCC
Kruskal-Wallis, p < 2.2e-16
3.7e-14
0.73
0.16
0.29
Probability of disease
1.5
1.0
0.5
0.0
Control
Cirrhosis
Com
Dec
HCC
Com
Dec
